# Overstatements and Understatements in the Debate on Synthetic Biology, Bioterrorism and Ethics

**DOI:** 10.3389/fbioe.2021.703735

**Published:** 2021-12-15

**Authors:** Anders Melin

**Affiliations:** Malmo University, Malmo, Sweden

**Keywords:** synthetic biology, ethics, risk, bioterrorism, precautionary principle

## Abstract

Synthetic biology has many valuable applications, but it also gives rise to certain risks. In this paper I discuss the risk of bioterrorism, which often attracts attention in both the mass media and scientific debate, as well as in government reports. While some authors argue that there is a significant risk of bioterrorism connected to synthetic biology, other scholars claim that the risk is exaggerated and that actors often have motives for overstating the risk. In this paper, I argue that some estimates of the risk may be overstated but that certain risks of bioterrorism, such as the creation and spread of known pathogenic viruses, need to be taken seriously. Actors may also have scientific and financial motives for understating the risk. Such understatements are sometimes based on a principle of hope, which says that technological progress is important for the future welfare of humanity and that too much precaution would have bad consequences. I argue that this principle is problematic as the burdens and benefits of synthetic biology may not be equally divided between different social groups. Instead, I claim that the principle of precaution is more justified as a point of departure for assessing advancements within synthetic biology. It tells us that we need strong evidence that such advancements are safe, because there is a potential risk that they may make it easier for terrorist groups to create and spread known pathogenic viruses.

## Introduction

Synthetic biology is an interdisciplinary research area within biology and engineering. It can be described as an umbrella term that denotes both new applications of previous forms of biotechnologies and new biotechnologies. It is a broad area with different subfields, such as synthetic genomics, bioengineering and xenobiology. Synthetic biology emerged as a new field of biotechnology in the early 2000s because of the convergence of biology and engineering principles. However, it is important to recognize that there is no exact divide between synthetic biology and earlier forms of biotechnology. Synthetic biology can be seen as part of the continuum of biotechnological progress that started with the development of recombinant DNA technologies, molecular cloning and polymerase chain reaction in the 1970s and 1980s (Keiper and Atanassova, 2020, 1–2). However, what distinguishes synthetic biology from earlier forms of biotechnology is that today’s scientists have greater capabilities to rationally design and construct biological systems and living organisms, which have many practical applications (Häyry, 2017, 187). For example, the creation of synthetic gene circuits enables the mapping of molecular origins of diseases and enhances drug discovery (Beitz et al., 2021).

Synthetic biologists design and construct biological systems and living organisms with the purpose of improving the applications for industry or biological research. Within synthetic biology technologies normally used within engineering and development are applied on living beings (Freemont and Kitney, 2016, 19–20). At present, synthetic biologists are not able to create fully artificial cells, but they have succeeded in creating synthetic genomes and transplanting them into cells. For example, a research group at J. Craig Venter Institute created in 2010 a synthetic genome for a bacterial species, *Mycoplasma mycoides*, and transplanted it into another bacterial species, *Mycoplasma capricolum*, thereby transforming the latter organisms into the former. The new organism was named “JCVI-syn1.0” or more informally “Synthia” (Powell, 2018). In 2016 a new version of the synthetic organism was presented containing only 473 genes, the minimum number required to sustain life under ideal environmental conditions (Sleator, 2016).

The creation of microorganisms with synthetic DNA can enable many valuable applications for humans, but it also involves certain risks. These risks have led some organizations to argue for a moratorium of some forms of synthetic biology, for example, in a report by Friends of the Earth and some other NGOs (International Center for Technology Assessment ETC Group, 2012). In the mass media and the scientific debate and in government reports it is often argued that the expanded capabilities to design and create living organisms lead to serious ethical challenges (Braun et al., 2019). One of the more spectacular challenges that is often highlighted and which I focus on here is the risk that terrorists make use of scientific results and technologies from synthetic biology to create dangerous bacteria or viruses. However, some scholars argue that risk assessments of bioterrorism often are exaggerated and that those who put forward such claims have motives for overstating their case. In this article, I discuss, based on the present scientific debate, whether we should take the risk of bioterrorism due to advancements within synthetic biology seriously and what the ethical implications would be.

## Overstatements and Understatements in the Debate

The mass media debate and the scholarly debate on synthetic biology, as well as government reports from both the US and the EU often highlight the increased risk of bioterrorism due to advances within synthetic biology (CBS News, 2021; Ahteensuu, 2017; National Science Advisory Board for Biosecurity, 2010; The European Group on Ethics in Science and the New Technologies (EGE), 2009. Some claim that synthetic biology may in the near future make it much easier for persons without long education and training to engineer new and dangerous forms of viruses and bacteria (Mukunda et al., 2009). However, the view that the advances within synthetic biology lead to significantly increased risk of bioterrorism is questioned by other authors, such as Jefferson et al., who argue that it rests on certain problematic assumptions. They claim that even if standardization and mechanization within the field has reduced the dependence on highly trained persons, advanced skills and technology and large infrastructures are still necessary. Moreover, the importance of tacit knowledge that one can only receive through extended lab training makes it very difficult to perform the necessary laboratory work without such training. In addition, it is much more difficult than often portrayed in the debate to design new dangerous pathogens with synthetic DNA, partly because viruses and bacteria cultured in laboratories often lose their virulence. Finally, even if a terrorist group would succeed in creating new dangerous pathogens, they also need considerable resources and knowledge to scale up the production of these pathogens, to store them and disseminate them efficiently. Jefferson concludes that scenarios of how individuals or groups without long training and considerable resources create microorganisms with synthetic DNA and transmit them efficiently is better understood as speculations about a distant future than predictions about what is possible at present (Jefferson et al., 2014, 1–12).

Jefferson et al. point out that actors in the mass media and scientific debate often have motives for overstating the risks of bioterrorism. For example, individuals in the security field may exaggerate the risks of bioterrorism to attract resources and attention. Moreover, they argue that exaggerated claims about the risks of synthetic biology sometimes are a result of exaggerated claims about its positive effects (Jefferson et al., 2014, 13).

The risk of bioterrorism due to the advancements within synthetic biology is also downplayed in an article by Søren Holm. He points out, first of all, that concern about bioterrorism is rather old, and has been voiced already in connection with much simpler technologies, such as culture of unmodified pathogenic bacteria. The additional risks identified in relation to synthetic biology are often related to the unrealistic assumption that persons without considerable training and resources would be able to create pathogenic organisms. Moreover, Holm argues that the consequences of bioterrorism are not likely to be as catastrophic as often assumed in the debate. Even if bioterrorism would kill an average of 100,000 people every year, it is doubtful whether it should be regarded as a major ethical issue as 842,000 people die from lack of access to clean drinking water every year. Holm concludes that the common exaggeration of the risks of bioterrorism within the bioethics literature is best understood as an attempt to support precautionary measures or to attract funding (Holm, 2017, 230–234).

Jefferson et al. and Holm seem right in claiming that there have been some exaggerations in the debate, but it still seems problematic to conclude that we should completely disregard the risk of bioterrorism due to advances in synthetic biology. I am not convinced of Holm’s argument that bioterrorism will kill less people than what die from lack of access to clean drinking water and therefore is not a major ethical issue. There are many other dangers in society that kill less people than what die from lack of access to clean drinking water, but which we still take seriously and with good reasons. The fact that we take these dangers seriously even though the problems of bad drinking water remain unsolved is better understood as a consequence of insufficient political will to solve the issue of access to clean drinking water, which arguably should be a prioritised issue.

We need to acknowledge that even though some risks connected with the production of pathogens based on advances within synthetic biology are very low, others may be higher. The risk of the production of completely new forms of pathogens seems very low at present, as it is almost impossible even for scientists with considerable time and resources to create such pathogens from scratch (Li et al., 2021, 1–14). Also some other risks are relatively low, for example, the risk that a terrorist group should succeed in making existing viruses more dangerous. It should also be pointed out that changes in a viral genome does not require advanced synthetic biology techniques, but only standard recombinant DNA technology. In addition, the risk that terrorist should be able to make existing bacteria more dangerous with the help of synthetic biology approaches is rather low. Although it is easier than creating completely new forms of bacteria, there are difficulties in scaling up the production. Moreover, re-creating known pathogenic bacteria involves large technical difficulties because of the large size of the bacterial genome (Gómez-Tatay and Hernández-Andreu, 2019, 1599; National Academies of Sciences, 2019). However, one risk that we probably need to take more seriously is the creation of known pathogenic viruses as it is relatively easy and inexpensive, and only requires access to basic laboratory equipment. Examples of pathogenic viruses that have been synthesized so far are the poliovirus, the Spanish influenza virus and the horsepox virus that is closely related to the smallpox virus (Gómez-Tatay and Hernández-Andreu, 2019, 1599; National Academies of Sciences, 2019).

One possible counterargument against the claim that we ought to take seriously the risk that terrorists create known pathogenic viruses is that they may be able to achieve their aims with much simpler means. This argument is put forward by Jefferson et al. who point out that the known examples of bioterrorism, such as the two failed attacks in Tokyo by the Japanese cult Aum Shinrikyo, have been conducted by using cultured bacteria, in the Aum Shinrikyo case *B. anthracis* (Jefferson et al., 2014, 12; Danzig et al., 2011). However, the fact that bioterrorists historically have not made use of advanced technology is not necessarily a valid argument for the conclusion that they will not do so in the future. It is often argued that it is easier today for terrorists to employ advanced technology, because of the increased spread of the required know-how, as well as increased availability of the necessary techniques and instruments (Ahteensuu, 2017, 1547–1552). Moreover, we cannot be certain that terrorists with the aim of impacting a large part of a population would not prefer to reconstruct and spread a pathogenic virus, rather than spreading cultured *B. anthracis*. Even if they risk being victims of the disease themselves, it may not necessarily discourage them, as some terrorists are willing to sacrifice their own lives to cause damage to others.

Although it seems correct that we need to recognize that individuals may have reasons for overstating the risk of bioterrorism due to the progress within synthetic biology, we should acknowledge that the opposite may also be the case. Both corporations and nation-states may have scientific as well as financial motives for avoiding a moratorium or too complicated and costly regulations. It has been pointed out in the scientific debate that some government authorities have downplayed the risks of synthetic biology to promote scientific development. For example, Matti Häyry argues that the report from 2010 on the ethics of synthetic biology by the Presidential Commission for the Study of Bioethical Issues is based on an optimistic view of science and does not take possible future risks seriously. The report concludes that the development of synthetic biology may proceed without first scrutinizing its potential dangers (Häyry, 2017, 197–199; Presidential Commission for the Study of Bioethical Issues, 2010). Häyry argues that the reasoning of the Presidential Commission rests on what is often labelled as “the hopeful principle,” which assumes that technological progress is important for the future welfare of humanity and that too much precaution would therefore have bad consequences, all things considered. Technological development should continue unless we know that it is unsafe (Häyry, 2017, 200).

From an ethical perspective, the hopeful principle seems problematic, especially in light of the fact that the burden and benefits of synthetic biology may not be equally divided between different social groups and different nation-states. Technological advancements have often profited the already rich the most while the poor have borne most of the burdens, and it has been argued that synthetic biology will probably have the same impacts (Takala, 2017, 241). An outbreak of a serious disease caused by bioterrorism is likely to have much more negative consequences for populations in the poor parts of the world, as these countries have much more limited healthcare capacity and less ability to buy and distribute vaccines. Therefore, even if a certain advancement within synthetic biology has the potential for making life much better for some parts of the global population, we have strong reasons to be cautious if it leads to risks of an outbreak of an infectious disease.

The precautionary principle that is mentioned in many policy documents on synthetic biology seems more justified as a point of departure for assessing the risk of bioterrorism. It is often argued in the scientific debate that it should be applied in the field of synthetic biology as our knowledge of the possible outcomes of the technological advancements are very uncertain (Wareham & Nardini, 2015; Holm, 2019). The key idea behind the precautionary principle is that we should not make any decisions to develop a certain technology that may have negative consequences before its safety has been scientifically secured. Compared to a more conventional way of thinking about risks, the burden of proof is shifted from the opponents of a new technology to its advocates (Häyry, 2017, 199).

According to stronger formulations of the precautionary principle, an action should be blocked if it may have harmful consequences. One problem with stronger formulations of the precautionary principle is that they require us to consider only the possible harmful consequences of a technology and to disregard the possible benefits. We should avoid actions with only small risks of limited harm, even if they have considerable benefits. Therefore, advocates of the precautionary principle often argue that a reasonable formulation of the principle needs to include an evidence-harm proportionality rule, saying that the strength of the evidence we need to conclude that something is safe should be proportionate to the severity of the potential harm (Wareham and Nardini, 2015, 119–120).

If we apply a precautionary principle that includes an evidence-harm proportionality rule, we are justified in prohibiting technological advancements within the field of synthetic biology if they will result in terrorists being able to create and spread known pathogenic viruses, even though the likelihood is low. Because the spread of such viruses may lead to considerable damage, we need strong evidence that such advancements are safe.

## Conclusion

Some scholars argue that the risk of bioterrorism due to advancements within synthetic biology is often exaggerated and that actors in the debate have motives for overstating their case. Although I agree that some exaggerations can be found in the debate, some risks, such as the risk that terrorists create and spread known pathogenic viruses, should not be regarded as negligible. Moreover, we need to recognize that there may be understatements of the risk of bioterrorism in the debate as well and that different actors may have motives for downplaying the risk. Understatements of the risk are sometimes based on the hopeful principle, which seems problematic given that the burden and benefits of synthetic biology would probably not be equally divided between different groups of people. Instead, a precautionary principle, including an evidence-harm proportionality rule, seems a better point of departure. This principle should make us cautious of advancements within synthetic biology if they risk making it easier for terrorists to create known pathogenic viruses. We need strong evidence that such advancements are safe.

## Data Availability

The original contributions presented in the study are included in the article/Supplementary Material, further inquiries can be directed to the corresponding authors.

## References

[B1] Ahteensuu M. (2017). Synthetic Biology, Genome Editing, and the Risk of Bioterrorism. Sci. Eng. Ethics 23, 1541–1561. 10.1007/s11948-016-9868-9 28074376

[B2] Beitz A. M. Oakes C. G. Galloway K. E. (2021). Synthetic Gene Circuits as Tools for Drug Discovery. Trends Biotechnol., 1–16. 10.1016/j.tibtech.2021.06.007 34364685

[B3] Braun M. Fernau S. Dabrock P. (2019). (Re‐)Designing Nature? an Overview and Outlook on the Ethical and Societal Challenges in Synthetic Biology. Adv. Biosys. 3, 1800326. 10.1002/adbi.201800326 32648715

[B4] CBS News (2021). West Point Biochemist Warns About Threat of Bioweapons. Available at: http://www.cbsnews.com/news/bioweapons-threat-synthetic-biology/ (Accessed October 22, 2021).

[B5] Danzig R. Sageman M. Leighton T. Hough L. Yuki H. Kotani R. (2011). Aum Shinrikyo: Insight into How Terrorists Develop Biological and Chemical Weapons. Washington, DC: Centre for a New American Security.

[B6] Freemont P. S. Kitney R. I. (2016). Synthetic Biology: A Primer. (Singapore: World Scientific Publishing).

[B7] International Center for Technology Assessment ETC Group (2012). The Principles for the Oversight of Synthetic Biology. Washington, DC: Friends of the Earth.

[B8] Gómez-Tatay L. Hernández-Andreu J. M. (2019). Biosafety and Biosecurity in Synthetic Biology: A Review. Crit. Rev. Environ. Sci. Technology 49 (17), 1587–1621. 10.1080/10643389.2019.1579628

[B9] Häyry M. (2017). Synthetic Biology and Ethics: Past, Present, and Future. Camb Q. Healthc. Ethics 26, 186–205. 10.1017/S0963180116000803 28361718

[B10] Holm S. (2019). Deciding in the Dark: The Precautionary Principle and the Regulation of Synthetic Biology. Ethics Pol. Environ. 22 (1), 61–71. 10.1080/21550085.2019.1581419

[B11] Holm S. (2017). The Bioethicist Who Cried “Synthetic Biology”: An Analysis of the Function of Bioterrorism Predictions in Bioethics. Camb Q. Healthc. Ethics 26, 230–238. 10.1017/S0963180116000827 28361720

[B12] Jefferson C. Lentzos F. Marris C. (2014). Synthetic Biology and Biosecurity: Challenging the “Myths”. Front. Public Health 2, 1–15. 10.3389/fpubh.2014.00115 25191649 PMC4139924

[B13] Keiper F. Atanassova A. (2020). Regulation of Synthetic Biology: Developments Under the Convention on Biological Diversity and its Protocols. Front. Bioeng. Biotechnol. 8, 1–20. 10.3389/fbioe.2020:0031010.3389/fbioe.2020.00310 32328486 PMC7160928

[B14] Li J. Zhao H. Zheng L. An W. (2021). Advances in Synthetic Biology and Biosafety Governance. Front. Bioeng. Biotechnol. 9, 1–14. 10.3389/fbioe.2021.598087 PMC8120004 33996776

[B15] Mukunda G. Oye K. A. Mohr S. C. (2009). What Rough Beast? Synthetic Biology, Uncertainty, and the Future of Biosecurity. Polit. Life Sci. 28 (2), 2–26. 10.2990/28_2_2 20205520

[B16] National Academies of Sciences (2019). Biodefense in the Age of Synthetic Biology. (Washington, DC: The National Academies Press). 10.17226/24890 30629396

[B17] National Science Advisory Board for Biosecurity (2010). Addressing Biosecurity Concerns Related to Synthetic Biology. (Washington, DC: U.S. Department of Health & Human Services).

[B18] Powell K. (2018). How Biologists Are Creating Life-like Cells from Scratch. Nature 563, 172–175. 10.1038/d41586-018-07289-x 30405232

[B19] Presidential Commission for the Study of Bioethical Issues (2010). New Directions: The Ethics of Synthetic Biology and Emerging Technologies. (Washington, DC: NIH).

[B20] Sleator R. D. (2016). JCVI-syn3.0 - A Synthetic Genome Stripped Bare!. Bioengineered 7 (2), 53–56. 10.1080/21655979.2016.1175847 27111400 PMC4879981

[B21] Takala T. (2017). Finding Hope in Synthetic Biology. Camb Q. Healthc. Ethics 26, 239–245. 10.1017/S0963180116000839 28361721

[B22] The European Group on Ethics in Science and the New Technologies (EGE) (2009). Ethics of Synthetic Biology. Brussels: European Commission.

[B23] Wareham C. Nardini C. (2015). Policy on Synthetic Biology: Deliberation, Probability, and the Precautionary Paradox. Bioethics 29 (2), 118–125. 10.1111/bioe.12068 24602164

